# Post-Covid-19 Education and Education Technology ‘Solutionism’: a Seller’s Market

**DOI:** 10.1007/s42438-020-00164-x

**Published:** 2020-07-13

**Authors:** Marko Teräs, Juha Suoranta, Hanna Teräs, Mark Curcher

**Affiliations:** 1 TAMK School of Professional Teacher Education, Tampere University of Applied Sciences, Tampere, Finland; 2 Faculty of Social Sciences, Tampere University, Tampere, Finland; 3 TAMK School of Business, Tampere University of Applied Sciences, Tampere, Finland

**Keywords:** Covid-19, Pandemic, Critical pedagogy, Education technology, Paulo Freire, Technologization, Postdigital

## Abstract

The Covid-19 pandemic and the social distancing that followed have affected all walks of society, also education. In order to keep education running, educational institutions have had to quickly adapt to the situation. This has resulted in an unprecedented push to online learning. Many, including commercial digital learning platform providers, have rushed to provide their support and ‘solutions’, sometimes for free. The Covid-19 pandemic has therefore also created a sellers’ market in ed-tech. This paper employs a critical lens to reflect on the possible problems arising from hasty adoption of commercial digital learning solutions whose design might not always be driven by best pedagogical practices but their business model that leverages user data for profit-making. Moreover, already before Covid-19, there has been increasing critique of how ed-tech is redefining and reducing concepts of teaching and learning. The paper also challenges the narrative that claims, ‘education is broken, and it should and can be fixed with technology’. Such technologization, often seen as neutral, is closely related to educationalization, i.e. imposing growing societal problems for education to resolve. Therefore, this is a critical moment to reflect how the current choices educational institutions are making might affect with Covid-19 education and online learning: Will they reinforce capitalist instrumental view of education or promote holistic human growth? This paper urges educational leaders to think carefully about the decisions they are currently making and if they indeed pave the way to a desirable future of education.

Historically, pandemics have forced humans to break with the past and imagine their world anew. This one is no different. It is a portal, a gateway between one world and the next. We can choose to walk through it, dragging the carcasses of our prejudice and hatred, our avarice, our data banks and dead ideas, our dead rivers and smoky skies behind us. Or we can walk through lightly, with little luggage, ready to imagine another world. And ready to fight for it. Arundhati Roy (2020)

## The Covid-19 Pandemic and the Push to Online Learning

The Covid-19 pandemic raging around the globe has caused large-scale institutional and behavioural ‘shock effects’ in various areas of human activity including education. The impact on learners is unprecedented: on 9 April 2020, there are over 1,500,000,000 students worldwide from primary to tertiary level who cannot attend school (UNESCO 2020). Due to massive and unexpected closures, affected countries and communities have been forced to seek quick fixes in different digital learning platforms (Jandrić 2020a). These rapid moves from classroom to online teaching have set aside the more profound questions related to national educational policies and theoretical grounds and premises. Current conditions of formal educational systems can be described using Philip Strong’s (1990) model of epidemic psychology consisting of three consecutive and overlapping epidemics: those of *fear*, *explanation*, and *action*. Strong uses ‘epidemic’ as a metaphor representing collective psychological reactions to an epidemiological crisis. The first aspect involves an epidemic of *fear* and opens up a question: How can the educational systems and individual learners cope with the exceptional situation?

The second aspect is an epidemic of *explanation* and moralisation: ‘People may be unable to decide whether a new disease or a new outbreak is trivial or whether it is really something enormously important. They swing backwards and forwards from one state of mind to another’ (Strong 1990: 254). At the same time, different actors in administrative positions provide their accounts of how to make sense of the situation and ensure the continuation of teaching and learning. Politicians are, of course, at the front line of educational policy-making, simultaneously setting restrictions and measures based on health experts’ assessments and constructing their official and authoritative narratives. Social media allow experts and novices to share their rational and irrational views with little in the way of moderation. Lockdowns affect students in multiple ways, reinforcing inequalities and putting them under social and psychological stress. Parents and custodians are affected too, and many of them come to realize, perhaps for the first time, the social purpose of the educational system and its power to structure everyday life.

The third aspect is an epidemic of *action*. It demonstrates how educational institutions and teachers across the world’s educational systems transfer their work from classrooms and lecture halls to digital platforms almost overnight. This quick transition has also revealed gaps and shortcomings in how online learning has or has not been adopted in educational institutions. Efforts at covering these gaps have created an influx of various kinds of support such as drop-in sessions, free webinars and blog posts, emergency policy documents (e.g. Doucet et al. 2020), and even lessons learned from earlier university lockdowns (Czerniewicz 2020). Perhaps more importantly, the situation has become a new market opportunity for commercial digital learning platforms providers.

Some forms of emergency online learning are being criticized for failing to adhere to sound pedagogical principles, best practices, and earlier research (Hodges et al. 2020). On social media, prominent experts have questioned reasons driving some individuals, organizations, and companies so eagerly towards providing guidance, pondering whether their motivation has been driven by market reasons (Siemens 2020). Others have noted the potential negative outcomes if educational technology quick fixes are implemented without balancing their consequences (Selwyn 2020; St. Amour 2020). Quickly jumping on board with learning platforms and online learning has also raised concerns about privacy and surveillance and the impact on students’ lives and human dignity (Harwell 2020).

In the moment of crisis, educational organizations should think carefully about their choices regarding online learning and education technology. These choices can potentially echo in the future as new relations of power and control, new forms of student inequity and inequality, and other unpredictable effects (Selwyn 2020). In order to mitigate possible negative impacts, educational organizations should leverage past knowledge of online learning as something that can be more varied than just a way to deliver information. Otherwise they are in danger of falling into the trap of classic Bourdeausian ‘misrecognition’ (see Bourdieu 1984), that is, into interpreting digital learning as self-evident and all-encompassing solution to the more profound problems of current mass education and institutionalized teaching and learning.

Online learning can take many different forms, including those pedagogically more innovative and engaging than commonly used processes of knowledge delivery and assessment. It can be informed and shaped by different education-philosophical and pedagogical underpinnings (Teräs and Kartoğlu 2017). Thus, online learning should not be seen as ‘one thing’ or a pedagogy in its own right.

Online learning is often understood as synonymous to content-driven self-study, where the advantages are limited to (a relative) independence of time and space. However, a digital learning environment which consists solely of textual files and lecture capture videos shared through a learning management system is very different from a digital learning environment that utilizes a situated online learning design such as the authentic learning framework (Herrington et al. 2010), which centralizes collaborative knowledge construction and complex, authentic learning. Furthermore, engaging teachers and students in the development, implementation, and use of education technology can affect how successfully technology can support meaningful teaching and learning (Bates and Sangrà 2011; Howland et al. 2011).

## Education Technology and Datafication: Seller’s Market

Long before the Covid-19 pandemic, critical researchers have noted the discrepancy between promises and improvements brought by education technology (Cuban et al. 2001; Cuban 2004; Selwyn 2010; see also Mertala 2019).

Corporations’ eagerness to bring computers and other digital devices to educational institutions began in the early 1980s. Businesspeople, computer enthusiasts, and politicians considered then, as they do now, that information and communications technology (ICT) is a magic bullet that improves teaching and learning, disciplines students as a skilful future workforce, and enhances democracy both in educational institutions and in larger society (Cuban 2004; Cuban and Jandrić 2015: 432). As Cuban has summarized:

Once schools were wired and equipment was in place, policymakers assumed, teachers and students would use the information technologies regularly in classrooms, and once computers were used regularly in schools, the desired outcomes, divergent as they were, would naturally follow. In short, access to technology would lead to instructional use, and use would lead to achievement of the goals (Cuban 2004: 20-21).

This has not happened: there is no evidence yet that teachers and students would have benefitted from using computers in terms of ‘student participation either in schools or as high school graduates engaged in their communities’ (Cuban and Jandrić 2015: 432). Additionally, critics have pointed out how chosen ontological bases, for example with ‘technology enhanced learning’, might limit critical discussion of education technology (Bayne 2015). With the rise of ed-tech companies, digital learning platforms have been criticized for redefining, simplifying, and reducing the concept of learning to better fit the education technology revolution narrative (Knox et al. 2020; Manolev et al. 2019). Among the reductions is the return of behaviourism in the new clothes of digital ‘machine behaviourism’ (Knox et al. 2020). Some platforms are reported to employ psychological behavioural management techniques and rankings to model student behaviour according to the system (Manolev et al. 2019). This begs a question whether these platforms actually support better learning and connects with recent discussions on ethics (Macgilchrist 2019; Slade and Prinsloo 2013) and the impact of datafication and surveillance (Jarke and Breiter 2019). Wider sociological impact of digital platforms and their links to market-driven platform capitalism are also prominent areas of critique (Srnicek 2017; Williamson 2020a, b).

For companies in various fields, behavioural data has become the most important asset to be monetized (Zuboff 2019). This is increasingly the case with digital learning platforms as well (Birch et al. 2020). Datafication of education is based on principles of business intelligence and using data for improving competitive edge. As Lycett (2013: 381, emphasis added) observes, ‘[t]he lines between enterprise and social intelligence are becoming increasingly blurred, as action from decision making is oriented at influencing people’s (future) behaviour. From an industry perspective, [business intelligence] is consequently seen as a fruitful foundation for *innovation, competition and productivity*.’ What provides the impetus for business intelligence is the increasing availability of data. To be maximally effective, this data has to be rich in volume (there must be large amounts of it available), velocity (there must be a high data flow rate), and variety (it must come from many sources) (Lycett 2013).

The grand narrative of datafication of education and the value proposition of learning analytics generally focus on philanthropic goals such as promoting student engagement, informing teaching practice, or personalizing learning (e.g. DeFreitas et al. 2015). Yet, its connections to the competitive ethos of business intelligence are unquestionable. In the case of education technology vendors and their growing influence in the education sector, the two are inseparably intertwined (see Williamson 2020a, b). It is not unrealistic to imagine that the boom of online learning during the Covid-19 pandemic could further strengthen and accelerate current developments in platform capitalism (Srnicek 2017), that is, harnessing online platforms as profit-generating engines functioning on the basis of collecting and using ever-increasing masses of data.

Technology and datafication of education are typically synonymized with progress and economic growth (Birch et al. 2020). The proponents of data mining argue that education generates growing volumes of data, so this data should be processed to improve education. The logical conclusion follows: having more data would be better (*volume*, *velocity*, *variety*). This conclusion warrants expanded forms of data collection (which translate into expanded markets for vendors), and the logic of unrestricted data flow becomes the starting point for improving education (Couldry and Yu 2018). Different wearable technologies and Internet of things (IoT) solutions are the naturalized next steps in this development, until all elements of the educational process are transformed into continuous data flows. These flows generate profit for ed-tech companies, but their usefulness to students and teachers is questionable. Nevertheless, these developments are ushered in with the neoliberal logic that individuals and educational institutions have an ethical responsibility to submit to datafication in the name of better education (Couldry and Yu 2018).

In recent years, many have raised important and unanswered questions about educational data ownership, access, and ethics, in addition to the role of student-centred development (Slade and Prinsloo 2013; Drachsler and Greller 2016; Corrin et al. 2019; Prinsloo 2019; Tsai et al. 2019; Teräs and Teräs 2019). Policies and ownership of data directly impact teaching and learning practices. Broughan and Prinsloo (2019) note that interactions with data are strongly shaped by the question who is allowed to use and interact with data and analytics and in what roles. For example, when usage of data is focused on assessment and evaluation of students, and to administrative processes, students are treated as ‘data objects’ (see Koopman 2019). This reduces student agency with their data and potentials for using data in support of their learning. Therefore, Broughan and Prinsloo (2019) argue that it would be more beneficial to reframe students as ‘data owners’ and partners in discussions about what data is collected, who will use collected data, and for what purposes. Also others have made attempts to reframe the use of data and analytics to promote student agency and educational equity (Tsai et al. 2019).

Educational institutions are increasingly quantifying their operations, which create a temptation of using data for surveillance rather than support (see Selwyn 2019). Indeed, continuous surveillance in education has become so deeply naturalized that the question of whether personal educational data should be collected in the first place is rarely asked. Data collection is increasingly built in the educational process as a basic requirement, leaving no room to question whether or not data should be collected at all (Couldry and Yu 2018). This is certainly true in the educational sector. Students and parents often need to consent to data collection policies in order to use applications and platforms presented by the educational institutions. Brushing away the data collection dilemma with a simple notion of consent is impractical and ignorant of the power structures that underpin the networked information society (Mai 2016). However, the ability to continuously monitor what students and teachers are doing is often emphasized by education technology vendors as a key selling point (Couldry and Yu 2018).

Data has been declared to become ‘the new oil’ (Arthur 2013) that fuels all walks of life, including ed-tech. Following Williamson’s (2020a, b) analyses of critical political economy, it can be concluded that business-driven development of digital learning technologies and platforms is not primarily aimed at improving learning and teaching but at making profit (see also Mirrlees and Alvi 2020). This is especially dangerous in the Covid-19 pandemic lockdown where educational authorities need to act as quickly as possible to keep the structures of learning and teaching running, thus maintaining the operation of societies at large. Under the circumstances, there is no time for detailed comparisons of digital learning platforms and to ponder broader and deeper social and educational visions of their uses. Resources of individual countries vary significantly in their capacity to cope with the emergency switch to online learning. This provides opportunities for ‘digital learning industries’ to make profit at the seller’s market. During the Covid-19 pandemic, similar phenomena are being observed at global marketplaces for medical masks and other protective equipment (Subramanian 2020).

The pandemic-stricken world is especially vulnerable to capitalist market mechanisms in various areas from healthcare and care for the elderly to education. Therefore, this is a crucial moment to critically reflect on the direction that education sector wants to take in the future and to question who should have the power to control its future. Technological choices are neither neutral nor do they affect only their immediate contexts of application. Due to socially constructed nature of technology (Selwyn 2010), technological choices made during the Covid-19 pandemic will impact micro-level teaching and learning experiences and meso-level organizational relations, to create wider and unpredicted macro-level societal impacts.

## Critique of ‘Disrupting Broken Education’

The catchphrase and an utopia has been over decades, if not for centuries, that technology will somehow ‘disrupt’ or revolutionize education (Selwyn 2010; see also Postman 1992). On the one hand, there is Bastani’s (2019) caricatural vision of techno-optimism (without techno-determinism) and its predecessors (see Fuchs 2020a: 18–19). On the other hand, the argument of broken education is based on the popular notion that education is somehow ‘broken’ so it should *and* can be fixed with technology (Williamson 2020b). Underlying argument is, to say the least, often ill-defined. Technical solutions aiming at increasing student engagement via automated feedback, personalization, predetermined learning paths, and other efficiency increasing strategies based on the generation and use of digital data are often used to signify ‘solutions’. However, what these individual aspects mean in relation to a human definition of learning is ill-defined.

During worldwide Covid-19-induced lockdowns, schools and teachers are using almost any available digital tools to ensure the continuation of teaching and learning. Short-term solutions are socially, pedagogically, politically, and economically necessary, yet the time of crisis is not the best moment for making long-term political plans and/or investments in educational technologies. What is needed instead is critical analysis of these matters.

Therefore, we must first reflect on the hypothesis of ‘broken education’. One of the critics, among others such as A.S. Neill (*Summerhill*, 1960), John Holt (*How Children Fail*, 1964), as well as Everett Reimer (*School is Dead*, 1971) and Paul Goodman (*Compulsory Miseducation*, 1973) (see Cuban and Jandrić 2015: 430), who have claimed that institutional education is broken, was Ivan Illich. Illich (1971) argued that in schools students learn obedience to authority and the hidden rules of capitalist consumer society, and therefore suggested that societies must be deschooled. By ‘deschooling’, Illich aimed at education that was organized communally and in a collaborative and dialogical manner in the same way as his Brazilian colleague Paulo Freire (2018). Illich and Freire have similarities, but they also have their differences. Perhaps the biggest difference is their basic understanding of modernity. Illich is supremely critical towards modern project at large, in all its political and institutional forms, including educational institutions. He especially criticizes modern technological rationality. Freire, on the other hand, does not criticize modern project as such, but its manifestations in modern capitalist societies which, as well as basic premises of education, must be changed into socialist societies (see Jandrić 2020a; Cuban and Jandrić 2015).

Whereas recent Illich- and Freire-spirited research offer relevant criticisms of broken *capitalist* education (see Fernback 2018; Fuchs 2020a; Jandrić 2017; McLaren and Jandrić 2020), there is also a strand of emerging criticism of ‘broken education’ that comes from the opposite ideological direction, that is, from entrepreneurial and pro-capitalist actors. This strand of criticism is popular among those seeking to promote and sell educational technologies. It is often referred to as the ‘Silicon Valley Narrative’, specifically the tendency to focus on the technological aspects of adoption rather than the human and learning (Weller 2015; Watters 2016; Hendrick 2018). It is an idea promoted in books such as *Disrupting class: how disruptive innovation will change the way the world learns* (Christensen et al. 2008), as part of a wider culture of technological disruption promoted by the ‘move fast and break things’ credo which seems to show little or no regard whether the broken ‘things’ are people. Sebastian Thrun, the computer scientist in the field of AI and co-founder of Udemy, writes: ‘Education is broken. Face it. It is so broken at so many ends, it requires a little bit of Silicon Valley magic.’ (cited in Wolfson 2013). These ideas are firmly bolted into the foundations of the neoliberal discourse of technological hype followed by loose talk about mystic ways of digital educational technologies to wonder cure the problems of (formal) education. These ideas, however, are mere ‘empty signifiers’ that point to non-existent objects without agreed-upon meanings.

We should also study the assumption that digital educational technologies offer quick fixes to every possible problem—without further investigation into their intertwining pedagogical, political, social, and individual consequences. Trying to fix education with technology, *technologization*, is closely connected to *educationalization*, neoliberal developments that transfer structural societal problems that are inherently political for education to solve (Labaree 2008; Simons and Masschelein 2008; Peters et al. 2019). Still, as Peters, Jandrić and Hayes (2019: 252) have noted, ‘[t]he idea that education can resolve the problem of technological unemployment is a political construction which has by and large failed to deliver its promise’. Indeed, as Birch et al. (2020) observe, there is increasing concern that technoscientific innovation is not solving, or even mitigating, societal challenges; instead, it has become subsumed into finance, and return on investment is triumphing over all other considerations, including pedagogical and ethical ones. As a result, ed-tech solutions may reinforce problems rather than fix them—a good example being the earlier discussed phenomenon of datafication and analytics-based applications that have been criticized for fostering an unjust, behaviour-based performative culture, decision-making based on decontextualized and potentially flawed data, surveillance and behaviour modification, and promotion of a worldview in which the way to success is grounded in neoliberal notions of competitive individualism (Manolev et al. 2019; Knox et al. 2020).

In the Covid-19 pandemic, the hypothesis of ‘broken education’ offers an opportunity to ed-tech businesses to sell untested solutions which sometimes have little to do with proper teaching and learning philosophies. Some ed-tech companies are now generously offering their services and products for free in the prospect of further sales. As these tools become rooted in teaching practice, it becomes difficult to go back. In addition, and more disturbingly, some of these tools employ login requirements and tracking cookies to capture and gather data that can be monetized in the future. This is a rising business model in technoscientific capitalism, where the development of useful technological products and services is less important than the ownership and control of assetized personal data (Birch et al. 2020).

In summary, the validity of the question, if society is broken, should naturally be derived from what we mean by being broken. Can it be derived theoretically, empirically or somehow else? If we look at the concept of ‘broken’ theoretically, we should first ask what ‘broken’ means, and what it means to different worldviews? If the worldview is capitalism, the society might not be broken after all, but education might be, as it could always more efficiently and effectively serve the capitalist society. If the worldview is based on ideals of holistic human growth, equality, and generally a good life for all individuals and the environment, something else, such as the *society*, could be what is broken.

If the idea of ‘broken’ is to be defined empirically, it could then be observed in the reactions and activities that have taken place in the educational sector during the Covid-19 pandemic. One of the main concerns across the globe has been whether students would graduate and whether their studies would continue uninterrupted. This is a perfectly valid concern, but the ways it is discussed and materialized has a somewhat eerie and factory-like tone in it. Emergency educational responses to the Covid-19 pandemic embody a sense of concern that the big conveyor belt of education has either slowed down or stopped and that the solution is to introduce more machines to keep it running. Another dominant theme in the pandemic lockdown is the financial crisis, and perpetuation of the Silicon Valley idea that education is not efficient enough in its ‘production’ of graduates, who are understood as resources of labour and consumers for the capitalist economy. Instead, education (still) uses some precious time to educate people in various aspects of life that seem to have value that is hard or impossible to define in non-instrumental terms.

## What Should Be Done?

Not long before the outbreak of the coronavirus, Williamson et al. (2019) outlined what they see as pressing issues in education technology research. They point that educational research needs to shift its focus from offering evidence of how technologies can solve existing problems towards new problems raised by education technology. Months before the pandemic outbreak, Selwyn et al. (2020a, b) called for the development of a critical ed-tech agenda and proactivity. Already before the pandemic, leading scholars have clearly articulated an urgent need for development of critical education technology research (see Suoranta and Vadén 2010; Bulfin et al. 2015; Jandrić 2017).

Months into the Covid-19 pandemic, this sense of urgency becomes stronger. In a recent editorial, Jandrić (2020b: 236) asks a crucial question: ‘which consequences will the Covid-19 pandemic have in regards to the environment, surveillance, worldwide rise of fascism, democracy?’ The pandemic has revealed how fragile the capitalist economy and education as its conveyor belt are. We can already see that the pandemic-induced crisis is affecting education in the shape of new contingency measures. It is likely that digital education platforms will be increasingly acquired and implemented due to their affordances for surveillance and educational management and to keep educational institutions running even in a moment of crisis. Within the capitalist worldview and instrumental understanding of education, such technologies indeed appear as the solution.

The seller’s market is about the future of education. Will post-Covid-19 education be shaped by visions of public good and designs of critically reflective and holistic learning, or will it be influenced by company interests for new markets (Selwyn et al. 2020a, b; Williamson 2020a, b)? At the moment, as critical theorist Henry Giroux (2020a) puts it, ‘[t]he coronavirus pandemic has pulled back the curtain to reveal the power of a brutal neoliberalism — and its global financial markets — in all of its cruelty.’ And, as he further states, ‘[t]he current viral pandemic cannot be discussed outside of the crisis of politics and education.’

While the pandemic lasts, it is important to envision possible futures. It is useful to exercise sociological imagination in possible ed-tech dystopias such as dataveillance and algorithms to control students (see Selwyn et al. 2020a, b; Macgilchrist et al. 2020), and it is just as important to envision and articulate what positive outcomes may look like. In addition to crisis talk (the language and pedagogy of critique), we thus need utopian visions and critical solutions (the language and pedagogy of possibility) which ‘shamelessly call a non-alienated, decent life’ (Žižek 2020: 114). The Covid-19 pandemic is not the end of *the* world, yet it could mean the end of *a* world of corporate greed in the pre-/context of predatory capitalism.

At the intersections of education and technology, Illich (1971) located the language of possibility and hope half a century ago in learning webs. Together with Freire, Illich believed that proper education is based on political literacy starting from interests and experiences of students. Post-Covid-19 world (or, perhaps more precisely, the world *with* Covid-19), demands creative, explorative learning implemented in open access peer-to-peer platforms where people ‘are able to meet around a problem chosen and defined by their own initiative (...) peers currently puzzled about the same terms and problems’ (Illich 1971: 19). Furthermore, the end of the world of corporate greed in the pre-/context of predatory capitalism cannot happen inside the authoritarian institutions of education but can be accomplished in learning webs. ‘The most radical alternative to school would be a network or service which gave each man (*sic*) the same opportunity to share his (*sic*) current concern with others motivated by the same concern’ (Illich 1971: 19).

Illich believes that there is an abundance of learning resources at our disposal outside formal schooling, but ‘they are neither conventionally perceived as educational resources, nor is access to them for learning purposes easy, especially for the poor’. Therefore, we need to ‘conceive of new relational structures which are deliberately set up to facilitate access to these resources for the use of anybody who is motivated to seek them for his education. Administrative, technological, and especially legal arrangements are required to set up such web-like structures.’ (Illich 1971: 78) These learning webs consist of, among other practices, skills exchanges and peer-matching that can even be facilitated by computers.

Illich’s ideals have been put forward in debates pertaining to open access and collaborative teaching and learning in the digital sphere. Suoranta and Vadén (2010) have suggested that the sphere of digital learning is a site for different social formations and political struggles. It

is built through the ‘collaborative turn’, or what is called participatory culture, which includes relatively low barriers to civic engagement and activism, artistic and other sorts of expression, easy access for creating and sharing one’s outputs with others, peer-to-peer relations and informal mentorship, as well as new forms of socialization, social connections, collectivism and solidarity. (Suoranta and Vadén 2010: 2.; see also Suoranta 2020)

In the world with Covid-19, we must be ready to oppose the looming ‘market-based language of profits, privatization, and commercial exchange’ (Giroux 2020b) in the area of educational technologies as well as taken for granted behaviourism and surveillance mechanisms in various digital learning solutions in the current seller’s market dominated by few digital barons. As Naomi Klein states,

we face real and hard choices between investing in humans and investing in technology. Because the brutal truth is that, as it stands, we are very unlikely to do both. The refusal to transfer anything like the needed resources to states and cities in successive federal bailouts means that the coronavirus health crisis is now slamming headlong into a manufactured austerity crisis. Public schools, universities, hospitals, and transit are facing existential questions about their futures. If tech companies win their ferocious lobbying campaign for remote learning, telehealth, 5G, and driverless vehicles — their Screen New Deal — there simply won’t be any money left over for urgent public priorities, never mind the Green New Deal that our planet urgently needs. On the contrary: The price tag for all the shiny gadgets will be mass teacher layoffs and hospital closures (Klein 2020).

If ed-tech indeed wins the game, we might be in danger, in C. Wright Mills’s term, of turning into ‘Cheerful Robots’—a tendency which, in its extreme, could lead to destruction of reason (Mills 2000: 170; see also Jones 2018). As Mills points out,

...in the extreme development the chance to reason of most men is destroyed, as rationality increases and its locus, its control, is moved from the individual to the big-scale organization. There is then rationality without reason. Such rationality is not commensurate with freedom but the destroyer of it (Mills 2000: 170).

At the very least, it can be said that the present ‘should be seen as complex and contested by a variety of forces’ (Kahn and Kellner 2007: 432), which leaves us with the open quest to find alternatives to technological determinism.

## Discussion and Conclusion

If we are to learn something valuable from Strong’s (1990) model of epidemic psychology, we should avoid repeating the three stages of social ‘epidemics’, *fear*, *explanation*, and *action*. Doing that, we avoid submitting ourselves to the same collective reactions to an epidemiological situation caused by Covid-19 that the model generally suggests. Otherwise, we are in danger of falling into an unreflective and perhaps faulty spin of fear, explanations (which can be mixed with disinformation, lies, and fake news), and action without possibility to surpass the dialectic of mere verbalism and activism. For, as Fuchs (2020b: 337) has pointed out, our ‘existential fears and needs can be instrumentalised’, and as a result, we might accept corporate ed-tech and other forms of domination as given.

During the Covid-19 pandemic, educational institutions have strived to find means to ensure students can continue their studies despite the crisis and social distancing. This has created an unprecedented push to online learning. In many cases, to ensure the continuation of studies, educational institutions have proceeded to find quick fixes with ed-tech. This has created a sellers’ market, where ed-tech companies have eagerly jumped on the opportunity to provide their services. Some have been willing to offer their services and platforms for free during the moment of crisis. Still, when looking at recent ed-tech platform developments, free might not be just for charity. For many current platforms, datafication, or leveraging user generated data for profit-making, has become the business model. This in mind, it is questionable whether many such service providers are there to develop better learning opportunities as such. Instead, the motivation may be to develop and optimize ed-tech platforms that leverage datafication as a business model. Getting their platform to use and users to generate data is therefore naturally their aim.

As discussed in this paper, the narrative of ‘education is broken and should (and can) be fixed with technology’ has a long history. Fixing every possible problem with technology, or *technologization*, is closely connected to *educationalization*, or imposing deeper societal problems and challenges for education to solve. Covid-19 crisis could also strengthen this increasingly Silicon Valley driven narrative of ‘disrupting education’.

It should be understood that ‘education is broken’ can mean different things when looked at from different worldviews. If education is seen as a conveyor belt to create graduates for the capitalist economy, then education might look like something to be fixed with technology in order for it to be more efficient. If education is seen as something that should promote holistic human growth for a more democratic and just society, leveraging digital technologies might look somewhat different: connecting people to discuss, learn, and tackle common problems together. Critical examination of technology makes us think what online learning should look like in the with Covid-19 world. If online learning is developed to support holistic human growth advocated by, e.g. Illich and Freire, it opens very different possibilities than developing it from an instrumental capitalist perspective. Therefore, this paper urges educational leaders to think carefully about the decisions they are currently making during the Covid-19 crisis and if they indeed are the best way to proceed for the future.

In order to develop a true *praxis* as critical intervention of our social surroundings and conditions, aiming at a human-centred world (Freire 2018; Fuchs 2020b: 338), we need to begin to imagine that a different world is possible and to act towards it. For, as Marx (1845) reminded us in his *Feuerbach Thesis* (#3), ‘[t]he coincidence of the changing of circumstances and of human activity or self-changing can be conceived and rationally understood only as *revolutionary praxis*.’ First, we should avoid the temptation to overcome the Covid-19 pandemic educational crisis with ready-made, top down solutions and ed-tech industry’s pedagogical colonialism. Secondly, the legacy of Illich, Freire, and other progressive thinkers urges us to join forces and start developing new educational practices horizontally and in a dialogical manner with our fellow teachers and other cultural workers.

This approach recognizes that the realm of digitalization is never neutral but one with a value dimension oriented towards objectives decided by human beings. It is important to be aware of those values, aims, and orientations that influence ed-tech decision-making. In Freire’s words, ‘[o]rientation in the world, so understood, places the question of the purposes of action at the level of critical perception of reality’ (1970: 206). In this perspective, the fundamental question is whether corporate-driven ed-tech is a dehumanizing structure causing teachers’ dependency. If so, then, as Freire says, ‘they cannot overcome their dependency by “incorporation” into the very structure responsible for their dependency’ (Freire 1970: 211). Instead they must replace, reclaim, or reinvent—or altogether abolish the dehumanizing structure.

An urgent task in the Covid-19 pandemic is to actively engage people, networks, projects, research, and public discussions to promote critically and reflectively informed praxis. We need to apply and develop critical applied research methodologies and create design principles for democratic and emancipatory digitalization of education. Moreover, we need wider societal dialogue about purposes of education and about the kind of society we want to develop in the with Covid-19 world. Technology and their providers should follow suit.
